# Proteasome subunit α4s is essential for formation of spermatoproteasomes and histone degradation during meiotic DNA repair in spermatocytes

**DOI:** 10.1074/jbc.RA120.016485

**Published:** 2020-12-04

**Authors:** Zi-Hui Zhang, Tian-Xia Jiang, Lian-Bin Chen, Wenhui Zhou, Yixun Liu, Fei Gao, Xiao-Bo Qiu

**Affiliations:** 1 Key Laboratory of Cell Proliferation & Regulation Biology, Ministry of Education and College of Life Sciences, Beijing Normal University, Beijing, China; 2 Medical Center for Human Reproduction, Beijing Chaoyang Hospital, Capital Medical University, Beijing, China; 3 State Key Laboratory of Stem Cell and Reproductive Biology, Institute of Zoology, Chinese Academy of Sciences, Beijing, China

**Keywords:** spermatogenesis, proteasome, α4s/PSMA8, meiosis, DNA damage, histone degradation, CP, core particle, DSB, DNA double-strand break, MSCI, meiotic sex chromosome inactivation, SYCP, synaptonemal complex proteins

## Abstract

Meiosis, which produces haploid progeny, is critical to ensuring both faithful genome transmission and genetic diversity. Proteasomes play critical roles at various stages of spermatogenesis, including meiosis, but the underlying mechanisms remain unclear. The atypical proteasomes, which contain the activator PA200, catalyze the acetylation-dependent degradation of the core histones in elongated spermatids and DNA repair in somatic cells. We show here that the testis-specific proteasome subunit α4s/PSMA8 is essential for male fertility by promoting proper formation of spermatoproteasomes, which harbor both PA200 and constitutive catalytic subunits. Immunostaining of a spermatocyte marker, SYCP3, indicated that meiosis was halted at the stage of spermatocytes in the α4s-deficient testes. α4s stimulated the *in vitro* degradation of the acetylated core histones, instead of nonacetylated histones, by the PA200-proteasome. Deletion of α4s blocked degradation of the core histones at DNA damage loci in spermatocytes, leading to meiotic arrest at metaphase I. Thus, α4s is required for histone degradation at meiotic DNA damage loci, proper progression of meiosis, and fertility in males by promoting proper formation of spermatoproteasomes. These results are important for understanding male infertility and might provide potential targets for male contraception or treatment of male infertility.

Proteasomes are responsible for degradation of most cellular proteins, and their inhibitors, such as bortezomib and carfilzomib, are clinically used to treat multiple myeloma and mantle cell lymphoma (1). Proteasomes usually contain one 20S catalytic core particle (CP) and one or two regulatory particles, which serve as activators, including the 19S regulatory particle, PA28α/β, PA28γ, and PA200 (2). The typical 26S proteasome contains the 19S regulatory particle and the 20S CP with constitutive catalytic subunits (including β1, β2, and β5) and promotes degradation of the ubiquitinated proteins. The immunoproteasome contains the 20S CP with the variants of catalytic subunits (including β1i, β2i, and β5i) (3). Certain fraction of 20S CPs in the PA200-containing proteasomes in testes harbors the catalytic subunits of the immunoproteasome, rather than regular catalytic subunits (4). The PA200-containing proteasomes promote the acetylation-dependent degradation of the core histones during somatic DNA repair and spermiogenesis (4, 5). In testes, proteasomes are largely specialized into spermatoproteasomes, which contain the testis-specific 20S subunit α4s/PSMA8 and/or the catalytic subunits of the immunoproteasome in addition to PA200 (4). α4s is specifically expressed in pachytene spermatocytes and the cells derived from them, including spermatids and spermatozoa (6).

Meiosis includes 2 cell divisions to produce haploid progeny. Spermatogenesis is a complex process in which primary spermatocytes progress through leptotene, zygotene, pachytene, and diplotene stages at prophase I of meiosis. After completion of meiosis I, secondary spermatocytes rapidly go through meiosis II to form haploid spermatids, which undergo spermiogenesis to differentiate into spermatozoa (7, 8, 9). During meiosis I, homologous chromosomes undergo genetic recombination by which DNA double-strand breaks (DSBs) are generated and then repaired, allowing them to exchange some of the genetic information. The subsequent repair of DNA DSBs is also critical to successful meiosis (10, 11). The X and Y chromosomes share homology only in a small segment, the pseudoautosomal region. This asynapsis leads to the prolonged DNA damage response. Thus, male sex chromosomes are associated with many DNA damage response proteins, including γH2AX (a phosphorylated form of the histone variant H2AX), at the XY body (12). The pseudoautosomal region forms DSBs at a higher frequency than typical autosome segments (11, 13). SPO11 makes DSBs through a topoisomerase-like reaction (12).

We demonstrate here that α4s/PSMA8 is required for the removal of the core histones at DNA damage loci, the proper progression of meiosis, and fertility in males by promoting formation of the properly assembled spermatoproteasome, which harbors both PA200 and regular constitutive catalytic subunits. During the preparation of this article, two independent works on the role of α4s/PSMA8 in male meiosis have been published (14, 15). Although they also demonstrated that the deletion of α4s/PSMA8 leads to male infertility in mice, the underlying mechanisms we each provide are complementary. Given that proteasomes are the known drug targets (1), our results might provide potential targets for male contraception or treatment of male infertility.

## Results

### Deletion of α4s leads to male infertility by stopping spermatogenesis at the stage of spermatocyte

**Figure 1:**
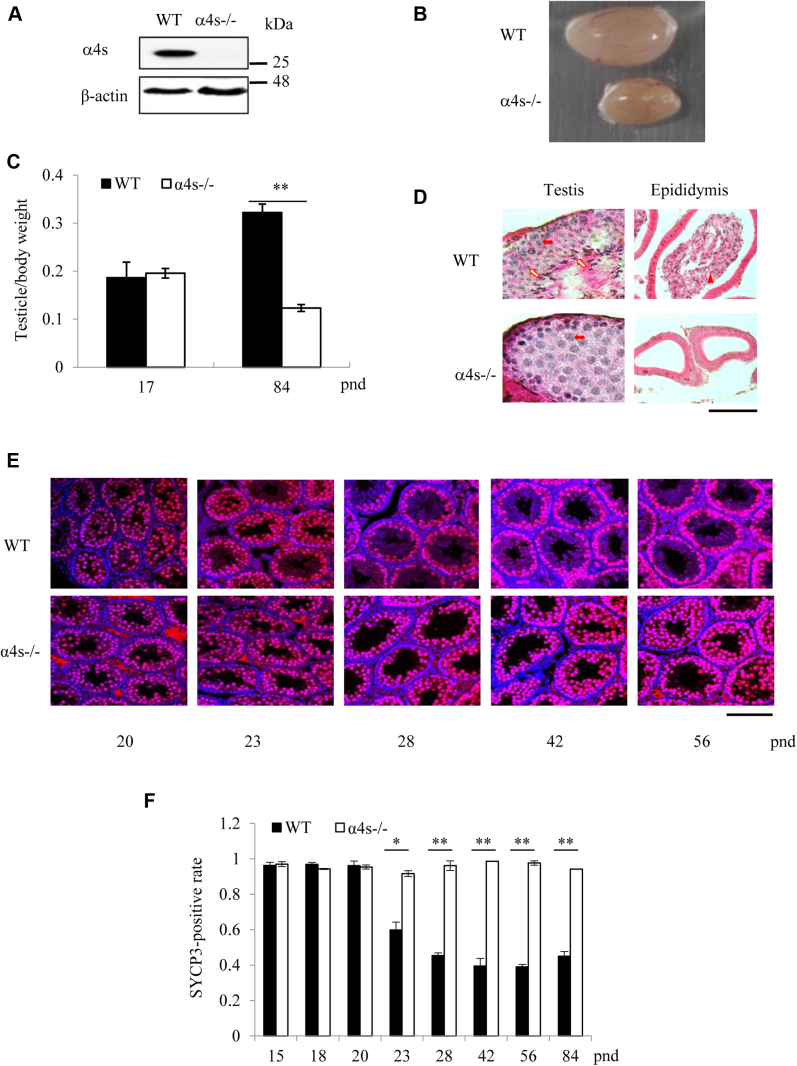
**Deletion of α4s leads to male infertility by stopping spermatogenesis at spermatocytes.***A*–*B*, immunoblotting analysis of α4s protein (*A*) and photograph of the testes (*B*) of the wildtype and the α4s-deficient mice. *C*, testicle weights relative to body weights of the wildtype and the α4s-deficient mice. *D*, H&E staining of histological sections of the testes and epididymis of the wildtype and the α4s-deficient mice. The *filled and open arrows* point to spermatocytes and spermatids, respectively, and the *triangle* points to a sperm. The scale bar represents 50 μm. *E*, immunofluorescent staining for SYCP3 in the paraffin sections of the testes of the wildtype and the α4s-deficient mice at various postnatal days (pnd). The scale bar represents 100 μm. *F*, percentage of SYCP3-positive spermatocytes was analyzed. Unless stated otherwise, all mice were 84 days old (mean ± SEM, n = 6). Data are representative of one experiment with at least two independent biological replicates. ∗*p* < 0.05, ∗∗*p* < 0.01 (two-tailed unpaired *t* test).

To investigate the role of α4s in spermatogenesis, we generated the mutant mice with global deletion of α4s gene (Fig. 1*A* and Fig. S1*A*). Homozygous deletion of α4s led to the reduced size and weight of testes in adult mice and caused male infertility but had no obvious adverse effects on female fertility or other male organs/tissues (Fig. 1, *B*–*C* and Fig. S1, *B*–*C*). There were few, if any, spermatids or spermatozoa in the seminiferous tubule and the epididymis from the α4s-deficient mice (Fig. 1*D*). Actually, a small fraction of haploid population (*i.e.*, 1C) of cells was observed in the α4s-deficient testes (Fig. S1*D*), suggesting that a relatively small number of spermatids survived after meiosis. Synaptonemal complex proteins (SYCP) 1, 2, and 3 are meiosis-specific scaffolds in spermatocytes (16, 17). Immunostaining of SYCP3 indicated that meiosis, which is not yet completed at postnatal day (pnd) 20, was halted at the stage of spermatocytes in the α4s-deficient testes (Fig. 1, *E*–*F*). As specifically marked by SOX9 (18), the formation of Sertoli cells (nurse cells) in testes was not affected by α4s deletion (Fig. S1*E*).

### Deletion of α4s reduces assembly of PA200 and regular catalytic subunits into proteasomes in adult testes

**Figure 2:**
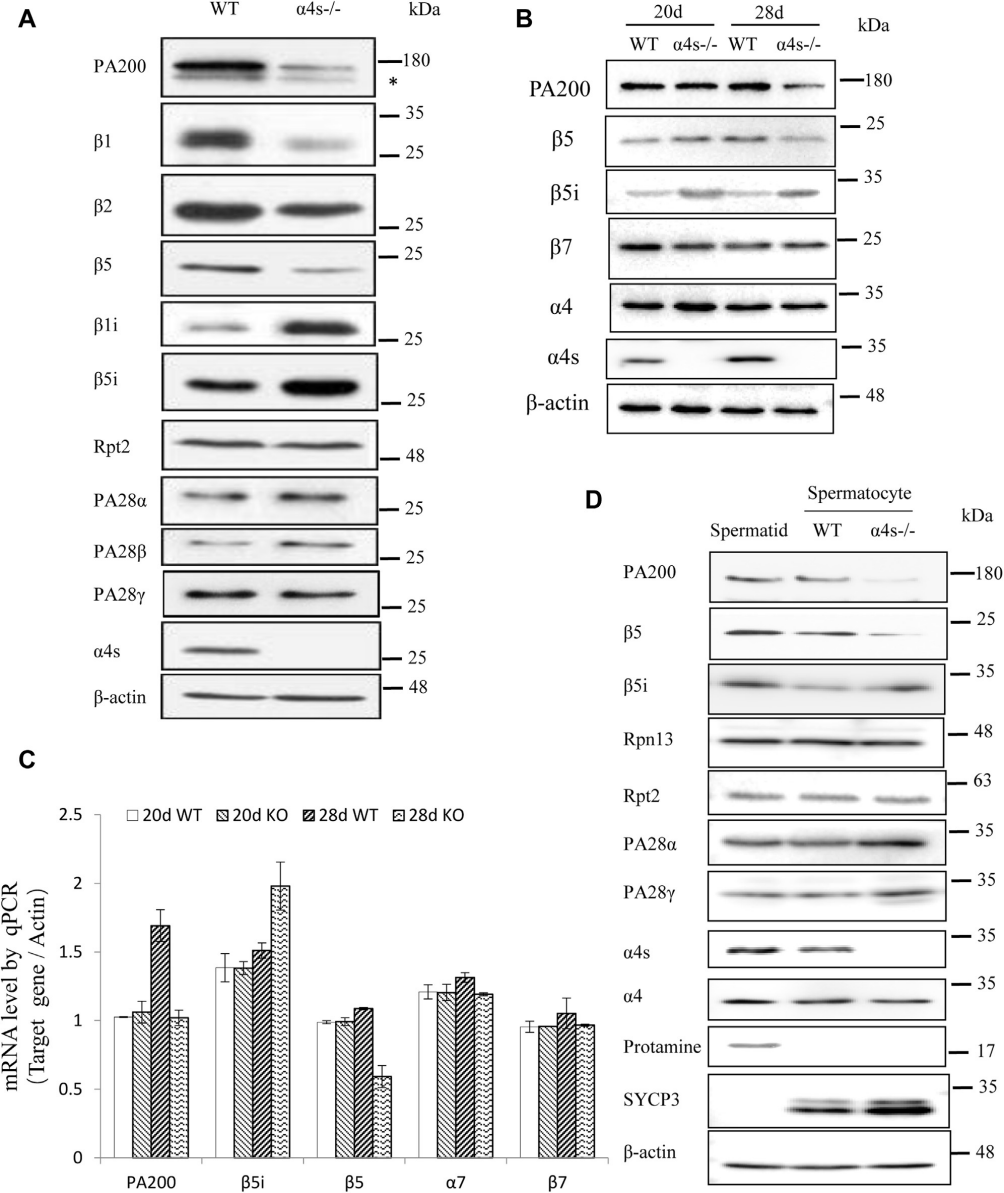
**Deletion of α4s reduces the amount of PA200 and regular catalytic subunits of proteasomes in spermatocytes.***A*, immunoblotting analysis of the extracts from the testes of the wildtype and the α4s-deficient adult mice. The *asterisk* indicates a nonspecific band. *B*, immunoblotting analysis of the extracts from the testes of the wildtype and the α4s-deficient mice at age 20 or 28 days. *C*, RT-PCR analysis of the mRNA levels of constitutive and immune-catalytic subunits and PA200. The mice were 20 or 28 days old. *D*, immunoblotting analysis of the extracts from the 4C spermatocytes of the wildtype and the α4s-deficient mice at age 28 days. Spermatid serves as a control. Data are representative of one experiment with at least two independent biological replicates (mean ± SEM, n = 6).

**Figure 3:**
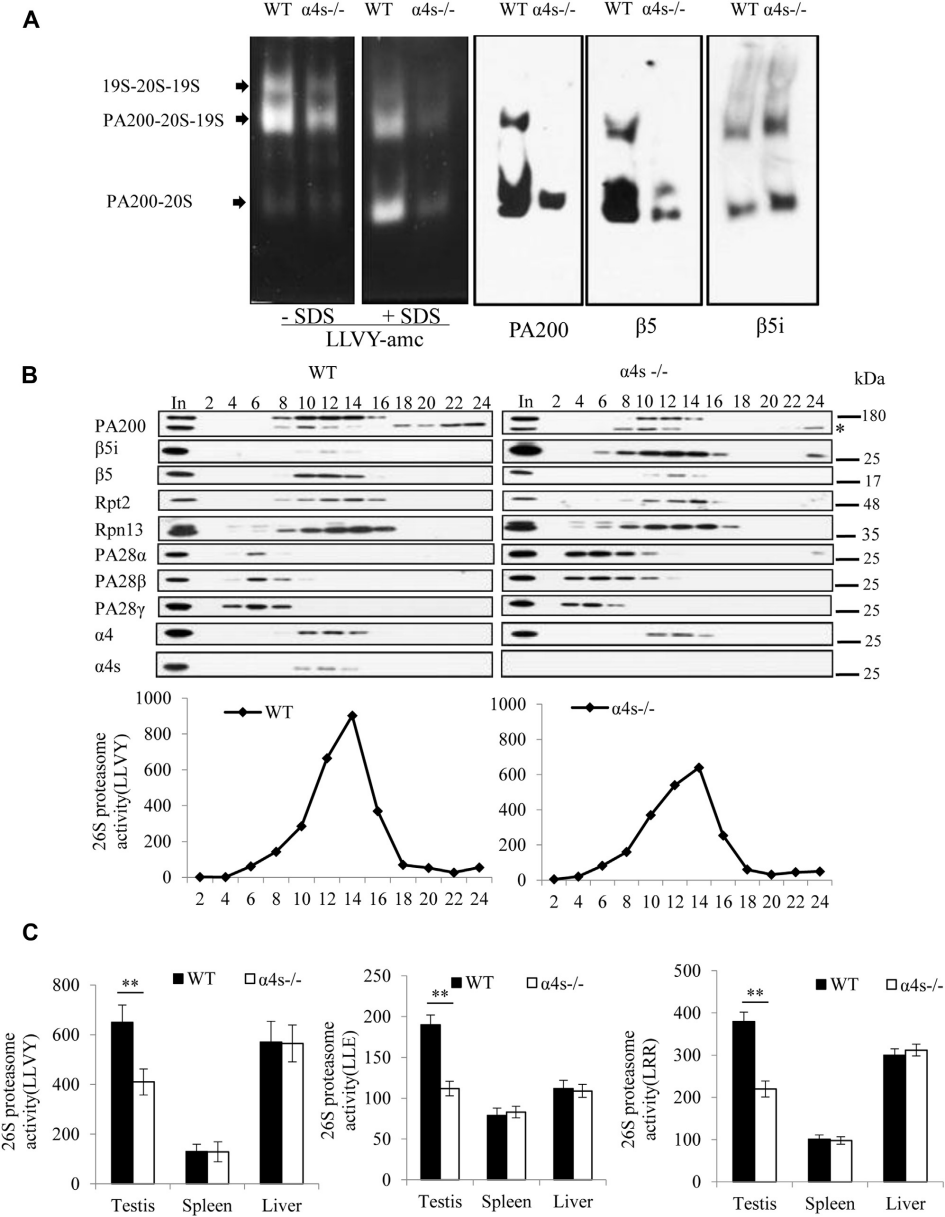
**Deletion of α4s reduces the amount of PA200 and regular catalytic subunits in proteasomes of mature testes.***A*, immunoblotting and peptidase activity analyses were performed following native PAGE of the extracts from the testes of the wildtype and the α4s-deficient mice. Proteasomal peptidase activity was analyzed by incubating the gel with LLVY-amc in the absence or presence of 0.02% SDS, which activates the 20S proteasome. *B*, immunoblotting of the fractions of glycerol gradient ultracentrifugation of the extracts from the testes of the wildtype and the α4s-deficient mice. Proteasomal peptidase activities were assayed using LLVY-amc as a substrate. *C*, the proteasomal peptidase activities of three tissue extracts of the wildtype and the α4s-deficient mice. All mice were 84 days old (mean ± SEM, n = 6). Data are representative of one experiment with at least two independent biological replicates. ∗∗*p* < 0.01 (two-tailed unpaired *t* test).

The typical 26S proteasome contains three constitutive catalytic subunits (*i.e.*, β1, β2, and β5) in addition to the 19S regulatory particle, whereas the immunoproteasome contains different catalytic subunits (*i.e.*, β1i, β2i, and β5i) (2). Deletion of α4s markedly decreased the protein levels of PA200 and all three constitutive catalytic subunits (*i.e.*, β1, β2, and β5) but increased the protein levels of the catalytic subunits of the immunoproteasome (*e.g.*, β1i and β5i) and the proteasome activators PA28α and PA28β in adult testes (Fig. 2, *A*–*B*). The protein levels of PA28γ and the other subunits from the typical 26S proteasome (*e.g.*, Rpt2, α4, and β7) had little, if any, change in the α4s-deficient testes (Fig. 2, *A*–*B*). To understand the mechanisms for these effects of α4s deficiency on the levels of various proteasome subunits, we demonstrated that the mRNA levels had similar changes to their protein levels (Fig. 2*C*), suggesting that α4s deficiency causes these changes in various proteasome subunits at least partially by altering their transcription or mRNA stability. Considering the difference in cell types, we purified 4C spermatocytes, which contain four times monoploid number of chromosomes (*i.e.*, 4C spermatocytes) at stages after DNA replication and before nuclear separation in the process of meiosis I, and haploid spermatids. Isolation of these types of cells was successful as indicated by the presence of protamine and SYCP3 (Fig. 2*D* and Fig. S1*D*), which mark spermatids and spermatocytes, respectively (16, 19). α4s deficiency had similar effects on the levels of the above-mentioned proteasome subunits in 4C spermatocytes in comparison with those in testicle homogenates (Fig. 2, *A* and *D*). Native PAGE and glycerol gradient analyses demonstrated that changes in the levels of these subunits were similar to those in the proteasomal complexes at or after pnd 28 (Fig. 3, *A*–*B* and Fig. S2*A*). Although a dramatic decrease in the levels of PA200 was not observed until pnd 28, replacement of constitutive catalytic subunits with immunoproteasome counterparts was obvious at pnd 23 in the α4s-deficient testes, as evidenced by the reciprocal changes in the levels of β5 and β5i (Fig. S2*A*). Using peptide substrates for proteasomes (*i.e.*, LLVY-amc, LLE-amc, and LRR-amc), we showed that deletion of α4s decreased all three peptidase activities of proteasomes in testes after pnd 23 but had no effect on these activities in young testes from mice at pnd 18, adult liver with the typical 26S proteasome, and adult spleen with the immunoproteasome (Fig. 3*C* and Fig. S2, *A*–*B*). Thus, α4s is required for the formation of the properly assembled spermatoproteasome, which contains both PA200 and regular catalytic subunits, in adult testes.

### Deletion of α4s increases the rates of spermatocyte apoptosis and disrupts MSCI

**Figure 4:**
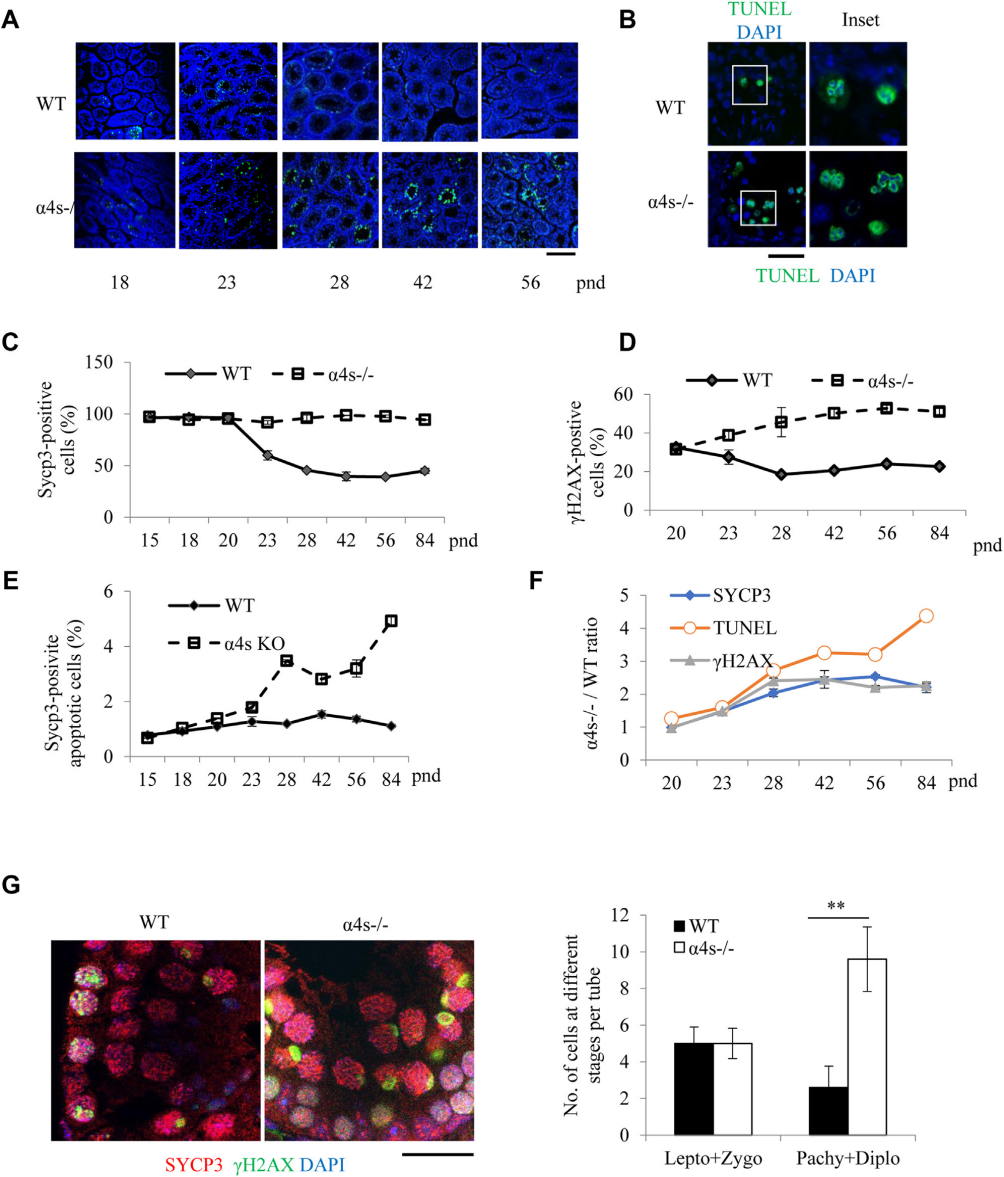
**Deletion of α4s increases the rates of spermatocyte apoptosis.***A*, TUNEL staining of the testicle sections of the wildtype or the α4s-deficient mice at different ages, visualized at low resolution (20×). The scale bar represents 100 μm. *B*, TUNEL staining of the testicle sections of the wildtype and the α4s-deficient mice at pnd 84, visualized at high resolution (100×). *C*–*D*, percentile of SYCP3- (*C*) or γH2AX (*D*)-positive cells in the wildtype or the α4s-deficient testes. *E*, percentile of apoptotic cells among the SYCP3-positive cells in the wildtype or the α4s-deficient testes. *F*, numbers of γH2AX-, TUNEL-, and SYCP3-postive cells in the α4s-deficient testes were normalized to those in the wildtype testes. *G*, immunofluorescent staining of the testicle sections of the wildtype and the α4s-deficient mice. γH2AX-positive spermatocytes were quantitated.

The TUNEL assay can usually detect apoptotic cells by attaching the fluorescently labeled nucleotides to the exposed 3’ ends of DSBs. Under low-resolution microscopy (20×) in the tissue sections, the TUNEL assay could show the condensed chromatin or apoptotic bodies, hallmarks for apoptotic cells. Deletion of α4s sharply increased the number of the apoptotic bodies-positive spermatocytes in the sections of testes (Fig. 4, *A*–*B*). Fluorescent annexin V conjugates provide reliable detection of the externalized phosphatidylserine, another indicator of apoptosis (20). Deletion of α4s also dramatically increased the number of the annexin V-positive spermatocytes in the sections of testes (Fig. S3, *A*–*B*). When normalized to that in the wildtype testes, the rate for the increased number of apoptotic spermatocytes was much higher than that for γH2AX- or SYCP3-positive cells during mouse development (Fig. 4, *C*–*F*).

SYCP1 and SYCP3 are present in autosomes only and all chromosomes, respectively. Deletion of α4s had not affected synapsis formation of either autosomes or sex chromosomes, which were differentially marked by SYCP1 and SYCP3 (Fig. S3*C*). In early pachytene spermatocytes, γH2AX is present only as small foci. In mid-pachytene to late diplotene spermatocytes, γH2AX is restricted solely to the XY body (21). Although deletion of α4s increased the number of the γH2AX-positive cells in testes (Fig. 4*G* and Fig. S4*A*), it did not affect the chromosomal distribution of γH2AX at various stages of prophase I of meiosis in spermatocyte nuclei (Fig. S4*B*).

Transcription in the XY body is repressed, leading to meiotic sex chromosome inactivation (MSCI) in the asynapsed sex chromosome regions, and sex chromosomes are depleted from active histone marks, especially acetylation (22). DSBs are required for this meiotic silencing. For example, mice with a mutation in *Spo11* are defective in MSCI (23, 24). Accordingly, *H2AX*-null male mice display meiotic arrest with MSCI failure (25). The X-linked gene, Hprt1, and the Y-linked gene, Rbmy1a1, are usually silenced during the MSCI in the wildtype testes (26) but were not silenced in the α4s-deficient testes. Their backup genes (Cetn1, Pdha2) located on autosomes are usually activated during MSCI but were silenced in the α4s-deficient testes (Fig. S4*C*). Thus, deletion of α4s disrupts MSCI, hinting an essential role of α4s in meiotic DNA repair.

### Deletion of α4s suppresses repair of DNA double-strand breaks at meiotic metaphase I

**Figure 5:**
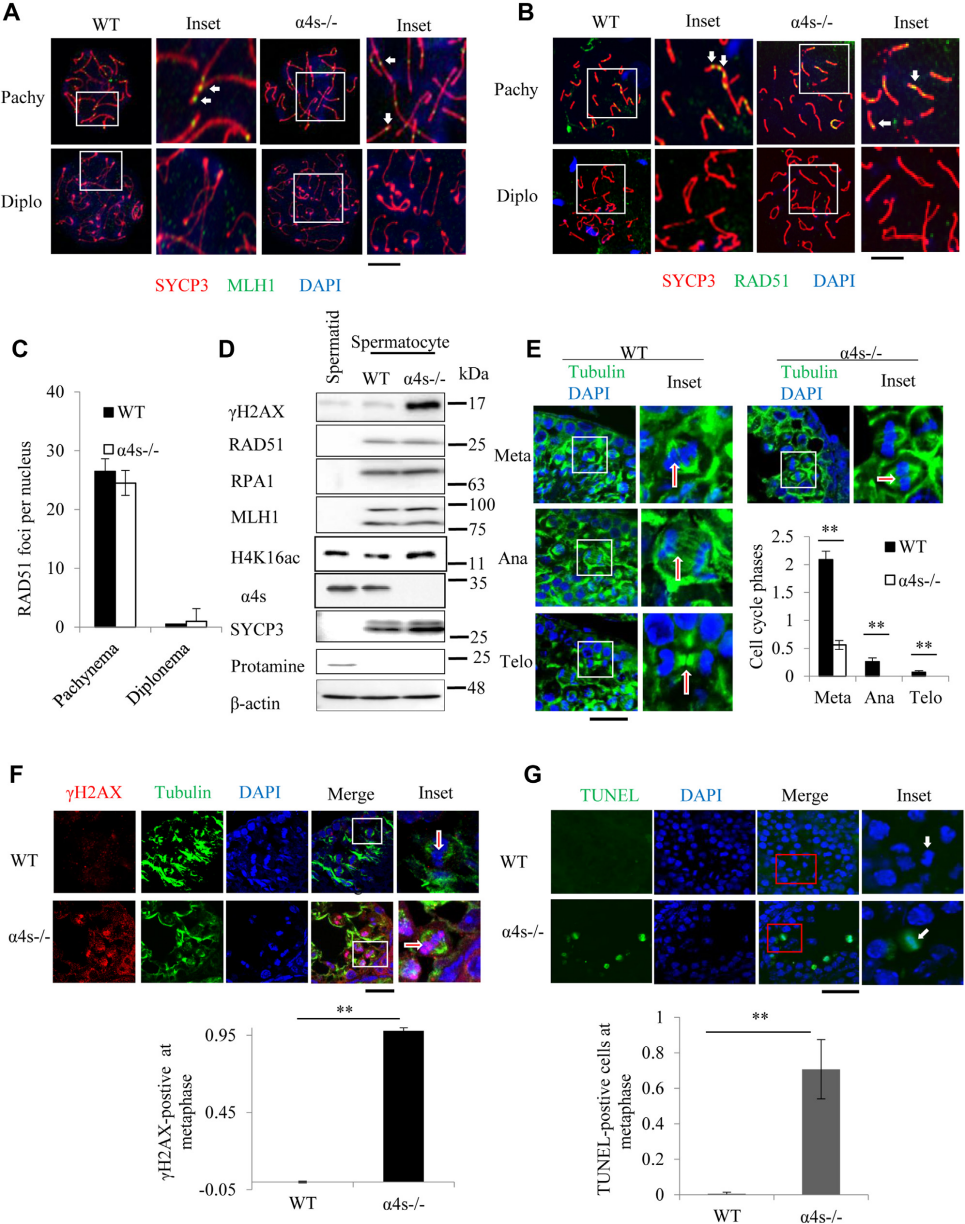
**Deletion of α4s suppresses repair of meiotic DNA double-strand breaks in testes.***A*, immunostaining of the spermatocyte nuclei from the wildtype or the α4s-deficient mice (n = 20). *White arrows* point to MLH1 foci. *B*–*C*, immunostaining of the spermatocyte nuclei from the wildtype or the α4s-deficient mice (*B*) and the numbers of RAD51 foci per nucleus were quantified (*C*). *White arrows* point to RAD51 foci. *D*, immunoblotting analysis of the extracts from the 4C spermatocytes of the wildtype and the α4s-deficient mice. Spermatids from the wildtype mice serve as a control. *E*, phases in meiosis I of the spermatocyte were examined in the wildtype and α4s-deficient testes by immunostaining of SYCP3 and α-tubulin. DNA was stained by DAPI. *White arrows* point to spermatocytes at corresponding phases. *F*, spermatocytes at metaphase I were examined in the wildtype and α4s-deficient testes by immunostaining of γH2AX and α-tubulin. DNA was stained by DAPI. *White arrows* point to spermatocytes at metaphase I. The scale bars represent 40 μm. *G*, TUNEL staining of the testicle sections of the wildtype or the α4s-deficient mice at pnd 28. TUNEL-positive cells at metaphase were quantitated. *White arrows* point to metaphase cells. The scale bar represents 40 μm. Except as stated in *G*, all mice were 84 days old (mean ± SEM, n = 6). Data are representative of one experiment with at least two independent biological replicates. ∗*p* < 0.05, ∗∗*p* < 0.01 (two-tailed unpaired *t* test).

Natural generation and subsequent repair of DNA DSBs are critical to mammalian meiosis and genetic diversity (10, 11). MLH1, a DNA mismatch repair protein, plays an important role in the formation of meiotic crossover in mid-pachynema (27). Deletion of α4s had no influence in the recruitment of MLH1, because the number of MLH1 foci did not change in the nuclei of the α4s-deficient spermatocytes (Fig. 5*A* and Fig. S5). Rad51 is required for heteroduplex formation in meiotic DNA repair and forms foci along the axial element from leptonema on. These foci are located along synaptonemal complexes in zygonema, and gradually disappear in pachynema (28, 29). In the α4s-deficient spermatocytes, the pattern for RAD51 foci was similar to that in the wildtype throughout prophase I (Fig. 5, *B*–*C* and Fig. S6, *A*–*B*), suggesting that deletion of α4s did not increase the recruitment of RAD51 onto chromosomes during prophase I.

To directly examine the role of α4s in meiotic DNA repair, we employed the TUNEL assay to monitor DSBs in the nuclei of nonapoptotic cells under a microscope with high resolution (100×) as reported (30). The numbers of TUNEL foci in the spread nuclei were similar between the wildtype and the α4s-deficient spermatocytes at prophase I (Fig. S6, *C*–*E*). In comparison with those in the wildtype testes, the levels of SYCP3 and γH2AX increased, but the levels of the acetylated histones, including H4K16ac, decreased, whereas the levels of the core histones (such as H2B, H3, and H4) did not change in the homogenates of the α4s-deficient testes (Fig. S6*F*). The decreased levels of acetylated histones in the α4s-deficient testes were apparently due to the disappearance of round spermatids, where acetylation of the core histones is known to be associated with histone displacement or degradation during the elongation of spermatids (4, 31). To exclude the influence of the cell types, we purified 4C spermatocytes and haploid spermatids. Indeed, deletion of α4s increased the levels of H4K16ac in the lysates of 4C spermatocytes in addition to the levels of SYCP3 and γH2AX (Fig. 5*D*, Figs. S6*F* and S7*A*). If DNA repair is normal, the levels of DNA repair proteins would increase, since the ratio of spermatocyte increased in the α4s-deficient testes (Fig. 1). However, the levels of MLH1, RAD51, and RPA1 all remained constant in both the homogenates of α4s-deficient testes and the lysates of 4C spermatocytes (Fig. 5*D*, Figs. S6*F* and S7*A*), hinting that deletion of α4s might block the repair of DNA breaks, resulting in eventual failure of meiosis and the ensuing apoptosis.

As revealed by staining of α-tubulin, deletion of α4s sharply reduced the numbers of spermatocytes at metaphase I and the ensuing phases, such as anaphase I and telophase I (Fig. 5*E*). γH2AX can mark chromatin domains with DNA breaks (32). Although there was no detectable colocalization between γH2AX and DNA at metaphase I in the wildtype testes, γH2AX colocalized with DNA in almost all spermatocytes at metaphase I in the α4s-deficient testes (Fig. 5*F*). In accord, deletion of α4s also caused the staining of DNA breaks by TUNEL assay at metaphase I of spermatocytes (Fig. 5*G*). These results suggest that deletion of α4s suppresses the repair of DSBs at metaphase I in spermatocytes.

### α4s is required for the programmed removal of the acetylated core histones during meiotic DNA repair

**Figure 6:**
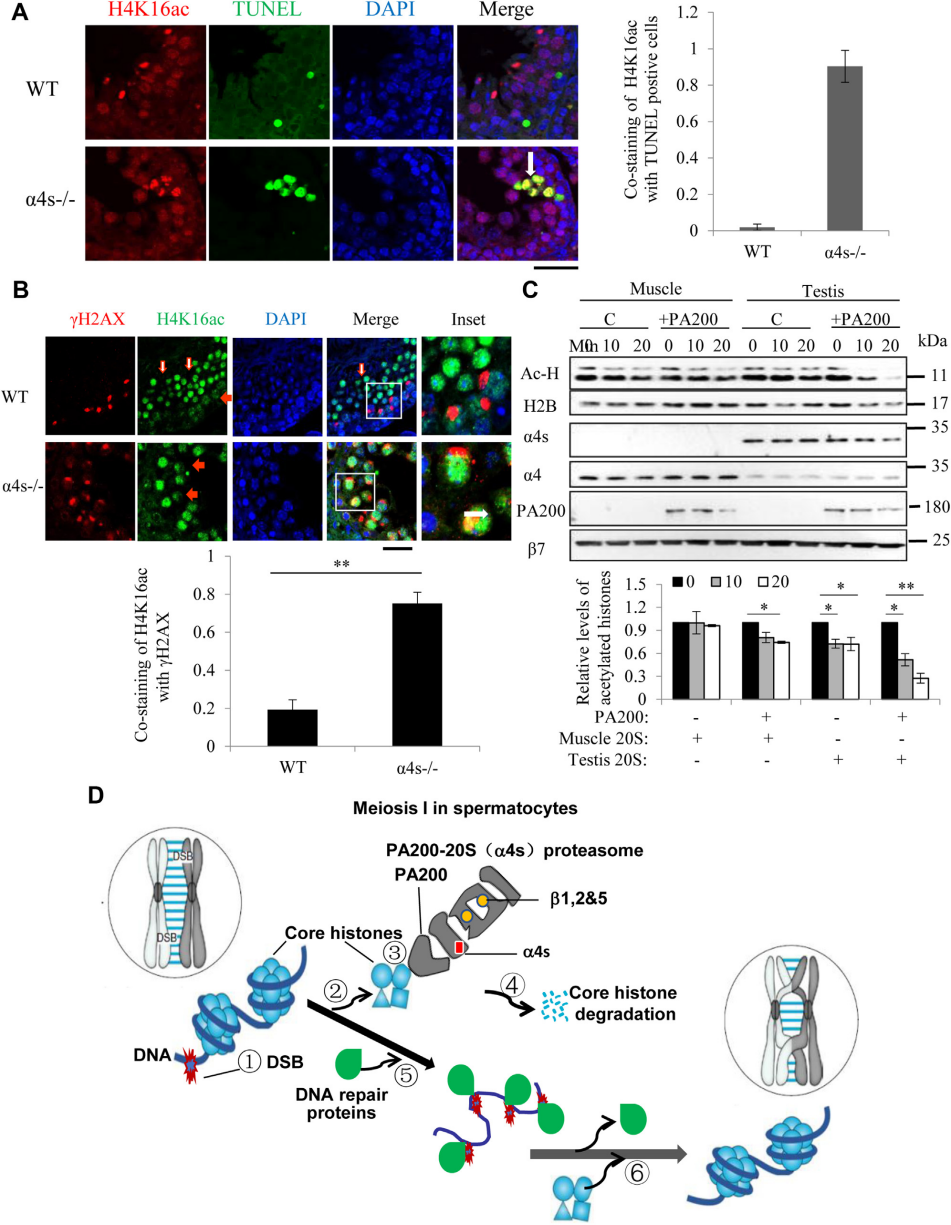
**α4s is required for programmed removal of the acetylated core histones in spermatocytes.***A*, colocalization of H4K16ac with DNA breaks marked with TUNEL staining was analyzed in the wildtype and α4s-deficient testes from mice at pnd 28 by immunostaining. The *white arrow* points to a spermatocyte with colocalization of TUNEL signal with H4K16ac. The ratios of cells containing H4K16ac to TUNEL-positive cells were quantified (mean ± SEM, n = 6). The scale bar represents 40 μm. Data are representative of one experiment with at least two independent biological replicates. ∗*p* < 0.05, ∗∗*p* < 0.01 (two-tailed unpaired *t* test). *B*, colocalization of γH2AX with H4K16ac was analyzed in the wildtype and α4s-deficient testes by immunostaining. The *filled and open red arrows* point to spermatocytes and spermatids, respectively, and the *white arrow* points to a spermatocyte with costaining of γH2AX with H4K16ac. All mice were 84 days old (mean ± SEM, n = 6). The scale bar represents 40 μm. *C*, immunoblotting analysis following the acetylation-dependent degradation assay for the core histones by the α4s-containg (from testes) and α4s-free (from muscle) 20S CP in the absence or presence of PA200. Degradation of the acetylated histones (Ac-H) was quantitated. *D*, model mechanism by which α4s promotes histone degradation, DNA repair, and meiotic progression in spermatocytes by stimulating assembly of spermatoproteasomes. Data are representative of one experiment with at least two independent biological replicates. ∗*p* < 0.05, ∗∗*p* < 0.01 (two-tailed unpaired *t* test).

Chromatins with meiotic DSB sites are sensitive to DNases in both yeast and mice, hinting that histones might be removed during meiotic recombination repair (33, 34). But, unlike that in the wildtype testes, H4K16ac partially colocalized with DSBs as marked by TUNEL staining and γH2AX in the α4s-deficient testes (Fig. 6, *A*–*B*), hinting that degradation of the acetylated histones was insufficient at DSB loci in the α4s-deficient testes.

Recently, we have demonstrated that the PA200-containing proteasomes degrade the acetylated core histones during somatic DNA repair and spermiogenesis (4). To test the role of α4s in histone degradation, we incubated the acetylated core histones with the 20S catalytic particles from testes, which contain α4s, in the absence or presence of PA200. The 20S particle from muscle, which does not contain any α4s, served as a control. α4s stimulated the *in vitro* degradation of the acetylated core histones, instead of nonacetylated histones (*e.g.*, H2B), by the PA200-proteasome (Fig. 6*C*). Finally, deletion of α4s suppressed the degradation of the acetylated core histones in testis lysates from mature mice (Fig. S7*B*). Taken together, our results suggest that α4s mediates degradation of the core histones during meiotic DNA repair, and is required for proper progression of meiosis and fertility in male mice.

## Discussion

The repair of DNA DSBs is critical to the completion of meiosis (10, 11). In mice, failure in meiotic DNA repair usually leads to arrest at meiotic prophase I (35) and eventually induces apoptosis (24). Even though meiotic DNA repair is not completed, chromosomes, including sex chromosomes, still remain paired before anaphase I in certain mammalian species (36). PA200 promotes the acetylation-dependent degradation of the core histones during somatic DNA repair and in elongated spermatids (4). A recent work suggests that the chromatin remodeler INO80 is required for the histone degradation coupled with DNA damage (37). This study shows that α4s is required for the removal of acetylated core histones during meiotic DNA repair in spermatocytes and male fertility. In comparison, deletion of PA200 just reduces the male fertility by delaying the degradation of the core histone during the elongation of spermatids after meiosis (4). Eventual cleavage of histones should be executed by catalytic subunits in the 20S particle. We suggested previously that spermatoproteasomes contain α4s/PSMA8 and/or the catalytic β subunits of immunoproteasomes in addition to PA200, because we had not presented the evidence whether or not spermatoproteasomes contain both α4s and the catalytic β subunits of the immunoproteasome (4). In this study, we show that α4s is required for the formation of the properly assembled spermatoproteasome, which contains both PA200 and constitutive catalytic subunits (*i.e.*, β1, β2, and β5), in adult testes. However, catalytic subunits of the immunoproteasome (*i.e.*, β1i, β2i, and β5i) could replace, at least partially, the constitutive catalytic subunits in the α4s/PSMA8-deficient testes. We further provide evidence that α4s deficiency causes the changes in the protein levels in various proteasome subunits at least partially by altering their transcription or mRNA stability. As a consequence, the phenotype of α4s deficiency was much more severe than that of PA200 deficiency. The switch between constitutive proteasome subunits and immunoproteasome subunits might also account for the severe phenotypes in the α4s/PSMA8-deficient mice. Thus, α4s should be a better drug target than PA200 in controlling histone degradation during spermatogenesis.

The difference between α4s and α4 is concentrated on the outer surface of the subunits (6). A large part of the surfaces generated by the unique regions of α4 and α4s are negatively charged. A remarkable difference is that the C-terminal unique regions have an opposite charge, *e.g.*, Glu223 for α4 and Lys225 for α4s (6). The extensive interactions between PA200 and the 20S catalytic particle result in significant α-ring conformational rearrangements (38). In 20S-PA200, the N-terminal tails of α5 to α7 of the 20S particle are fully ordered and relocated from the proteasome axis into grooves at the inner surfaces of the PA200 dome, but the tails are disordered and not recovered for the remaining α subunits, particularly evident for α3 and α4 (38). The difference in α4s sequence might make it a better fit than α4 for the assembly of the 20S particle with PA200, and perhaps for the recruitment of regular catalytic subunits, including β1, β2, and β5, in comparison to their immunoproteasome counterparts.

During the preparation of this article, two independent works on the role of α4s/PSMA8 in male meiosis have been published (14, 15). Zhang *et al.* (15) showed that the levels of DNA damage repair proteins RAD51 and RPA1 in mature testes were markedly elevated by the deletion of α4s/PSMA8 as analyzed by immunoblotting, whereas Go’mez-H *et al.* (14) showed no difference in the accumulation of DNA damage repair proteins RAD51 and MLH1 between the wildtype and the α4s/PSMA8-deficient spermatocytes as analyzed by immunostaining. In order to avoid the influence of the cell types, we analyzed the levels of these DNA damage proteins in the purified 4C spermatocytes by immunoblotting and further validated these results by immunostaining of spermatocytes. Our results demonstrate that the levels of DNA damage repair proteins RAD51, MLH1, and RPA1 were not elevated in the α4s/PSMA8-deficient spermatocytes, which were in accord with those by Go’mez-H *et al.*, clarifying this inconsistence in the literature. Although the levels of DNA damage repair proteins were not elevated in the α4s/PSMA8-deficient spermatocytes, we showed that deletion of α4s led to accumulation of unrepaired DNA breaks as marked by γH2AX and TUNEL staining at metaphase I, suggesting that α4s is essential for meiotic DNA repair and the proper progression of meiosis. Although two other papers also demonstrated that the deletion of α4s/PSMA8 leads to male infertility in mice, the underlying mechanisms in their papers are different from ours (14, 15). Although the report by Zhang *et al.* did not provide any valid candidate substrates for α4s/PSMA8, Go’mez-H *et al.* showed that other meiosis-related proteins, including SYCP1, SYCP3, CDK1, and TRIP13, in addition to the acetylated histones are also accumulated in the α4s/PSMA8-deficient spermatocytes. But they did not provide any evidence that these proteins are degradable by the α4s/PSMA8-containing proteasomes, and hence, they did not prove that these proteins are the substrates of the α4s/PSMA8-containing proteasomes. However, we found that α4s/PSMA8 directly promotes the proteasomal degradation of the acetylated histones in the presence of PA200, proving that the acetylated histones are the substrates of the α4s/PSMA8-containing proteasomes.

Taken together, our results suggest that α4s/PSMA8 promotes proper formation of spermatoproteasomes, which harbor both PA200 and constitutive catalytic subunits, and that α4s/PSMA8 is essential for DNA repair at metaphase I, proper progression of meiosis, and fertility in male at least partially by promoting histone degradation. We suggest that spermatoproteasomes are defined to be composed of the activator PA200 and the 20S catalytic particles with α4s as well as constitutive catalytic subunits (*i.e.*, β1, β2, and β5). As proposed in Figure 6*D*, at the meiosis I in spermatocytes, (1) DSBs are generated for exchanging genetic information between nonsister chromosomes, and the core histones near DNA damage sites are heavily acetylated; (2) the acetylated core histones are recognized by the BRD-like region in PA200 in the spermatoproteasome; (3) the subunits of the spermatoproteasomes, including PA200 and constitutive catalytic subunits, are expressed and assembled in the presence of α4s; (4) the acetylated core histones are degraded by spermatoproteasomes; (5) DSBs are exposed and repaired by DNA repair enzymes; and (6) the newly synthesized core histones are reassembled into chromatins. Thus, deletion of α4s suppressed the degradation of acetylated core histones, blocked DNA repair at metaphase I, disrupted MSCI, and finally led to male infertility. Although we could not exclude the involvement of other potential substrates of the PA200-containing proteasomes, degradation of the core histones during meiotic DNA repair in spermatocytes must play an important role in these α4s-related activities.

Errors in autosomal synapsis are usually associated with the unrepaired DSBs and asynapsis, which eventually lead to infertility (39). The failure of MSCI, which reverts the silencing of genes on X and/or Y chromosome, results in spermatocyte apoptosis during the pachytene stage (40). It has been reported that mutations in genes involved in MSCI, such as *H2ax* and *Brca1*, cause meiotic arrest (25, 41). Thus, the results from this study are important for understanding male infertility and might provide a clue for the treatment of male infertility.

Unplanned pregnancies, especially in teenagers, contribute to social and financial burden associated with abortions and deliveries by single mothers. Although there is a short list of possible targets for contraceptive drugs (42, 43, 44), reversible male contraceptive drugs are not yet available. Identification of the testis-specific α4s as an essential proteasome subunit for meiosis in male might provide a valid target for male contraceptive drugs. Since deletion of α4s does not affect spermatogenic cells upstream of pachytene spermatocytes, removal of the drug that targets α4s should resume the fertility.

## Experimental procedures

### Construction of the α4s-deficient mice

The α4s/PSMA8 mutant ES cells were generated in an ES cell line of the C57BL/6N mouse using the retroviral gene trapping techniques by the European Conditional Mouse Mutagenesis Program (EUCOMM, clone ID: EUCE0019_F06) and were then injected into C57BL/6N blastocysts. The resulting male chimeras were mated with female C57BL/6N mice. Heterozygous offspring were intercrossed to produce homozygous mutants. Mice were kept in School of Brain and Cognitive Sciences, Beijing Normal University, using standard humane animal husbandry protocols. The animals’ care was in accordance with institutional guidelines. Unless stated otherwise, mice were six per group and age- and sex-matched in each experiment. Sample size was based on empirical data from pilot experiments. No additional randomization or blinding was used to allocate experimental groups. Insert site in Chromosome 18 was 14870771, and the Sequence between 14870771 and 14870997 was the target of alignment (GAACAATTTGTTTTCTCTGCCAGATCAGCAATGTGCCTTCATTAGTGTCATCTATTCTCAGGATTACCTGAAGCGTTATGTGTAAGGGTGGTCTGATTTGGATGCCATGTTGATTTCTCTGGTGAGCAATAAGTAACAAGTTCTGTAGACACTTTGACAAGGTACATGTATGATTGAAAAATATTAACCCCACTAAAATTTAGAGTGCAAAATTCTGGTAAGTTTCT). For genotyping, DNA was extracted from the tip of the tail and analyzed by PCR with the primers as follows:

Forward primer: 5′-CAACCAGTATTATAGTGACCCAGC;

Reverse primer: 5′-GGGACTAGACTGTAGTACATTTGAGG.

### Immunoblotting

Unless stated otherwise, testes were homogenized in the buffer (50 mM of Tris-HCl [pH7.5], 150 mM of NaCl, 10% glycerol, 5 mM of MgCl_2_, 5 mM of ATP, and a protease inhibitor mixture) using a mortar, sonicated twice at 200 W for 10 s each, and then cleared by centrifugation. Proteins were separated by SDS-PAGE. After proteins were transferred to a polyvinylidene fluoride membrane (Millipore), the blot was incubated with a primary antibody. The secondary antibody was goat antibody against rabbit or mouse IgGs conjugated to horseradish peroxidase. To obtain whole tissue extracts, testes were triturated and incubated with cold acetone overnight. The extract was centrifuged and dried in air, and then the pellet was dissolved in 1.2× SDS sample buffer.

### Proteasome activity

Proteasome activity was analyzed by using peptide substrates, including LLVY-amc, LLE-amc, and LRR-amc, as described previously (45).

### Tissue collection and immunostaining

Testes were fixed in 4% paraformaldehyde at 4 °C overnight, dehydrated, embedded in wax, and sectioned at 5 μm. The sections were deparaffinized, rehydrated, and followed by antigen retrieval in 10 mM of the sodium citrate buffer. Then, sections were blocked with goat serum in 0.3% Triton X-100 and incubated with primary antibodies.

### H&E staining

Spleens and kidneys were excised and fixed in 4% paraformaldehyde overnight and sectioned at 5 μm and stained by hematoxylin and eosin. The cytoplasm was stained by eosin (red), and the nucleus was stained by hematoxylin (blue).

### Purification of spermatocytes and spermatids by flow cytometry

Testis dissociation was based on a recently described method (46). Testes were isolated, decapsulated, and incubated in 0.5 mg/ml of collagenase/DNase I/Dulbecco's modified Eagle's medium. The tube was shaken in a horizontal position at 150 rpm for 10 min at 35 °C, and then seminiferous tubules were incubated in preheated collagenase I/Dnase I/trypsin/Dulbecco's modified Eagle's medium. The tubules were gently pipetted up and down. The suspension was passed through a 100-μm nylon cell strainer and washed with 1x PBS. The cells were incubated with Hoechst and propidium iodide, respectively. The cells were then sorted by a flow cytometer (BD FACSAria III) and analyzed using BD FACSDiva.

### Apoptosis detection by TUNEL assay or annexin V staining

Apoptosis detection in the testes using the DeadEnd Fluorometric TUNEL System was carried out according to the standard paraffin-embedded tissue section protocol (Promega).

For annexin V staining of apoptotic cells, the sections were deparaffinized and rehydrated, followed by antigen retrieval in 10 mM of the sodium citrate buffer. Then, sections were blocked with goat serum in 0.3% Triton X-100 and incubated with primary antibodies of annexin V (Abcam, ab14196, 1:50).

### Spermatocyte spread and immunolabeling

Spreading and immunolabeling of testicular samples were performed according to the standard protocol (47). Briefly, testes were dissected, rinsed in PBS, and decapsulated. The remaining tissues were transferred into a separation medium (hypo extraction buffer containing 30 mM Tris pH8.2, 50 mM sucrose, 17 mM citric acid, 5 mM EDTA, 2.5 mM DTT, and 1 mM PMSF) for 30 min. Spermatocytes were released from the tubules by finely mincing with a razor blade in 0.1 M of sucrose solution. Twenty microliters of the mixture was added onto a glass slide preloaded with 500 μl of 1% paraformaldehyde (pH 9.2) and spread evenly. Slides were incubated in a humidified chamber for 2 h. For immunostaining, slides were blocked in 1× ADB (1% goat serum, 3% BSA, 0.2% Triton X-100) at room temperature for 10 min, followed by incubation with primary antibodies at 4 °C overnight. On the following day, slides were washed and incubated with secondary antibodies for 1 h and finally mounted with DAPI (1:200). Antibodies against SYCP1 (Abcam, ab15087, 1:100), SYCP3 (Abcam, ab97672, 1:100), MLH1 (Abcam, ab92312, 1:50), γH2AX (Millipore, 05-636, 1:50), and RAD51 (Abcam, ab133534, 1:100) were used.

### Purification of PA200 and the 20S proteasome

Purification of proteasomes from bovine or rabbit tissues was carried out as described (45). Purification of PA200 was adapted from previous protocols (4, 48). Briefly, the supernatant from the homogenized bovine testes (200 g) was incubated with DE52 DEAE cellulose (100 ml). The resin was washed with the buffer containing 20 mM of Tris-HCl (pH7.5), 10% glycerol, 50 mM of NaCl, 5 mM of MgCl_2_, 0.5 mM of EDTA, 1 mM of dithiothreitol (DTT), and 2 mM of ATP and was then eluted with 250 ml of the above buffer, but containing a 50 to 300 mM NaCl gradient. The pooled fractions with PA200 were diluted with an equal volume of the TSDG buffer (10 mM of Tris, pH 8.5, 25 mM of KCl, 10 mM of NaCl, 5.5 mM of MgCl2, 0.1 mM of EDTA, 1 mM of DTT, and 10% glycerol) and were loaded to Q Sepharose ion-exchange Fast Flow column. The column was rinsed with 20 ml of the TSDG buffer, eluted with 50 ml of 750 mM of KCl in the TSDG buffer, and the eluted sample was directly applied to the equilibrated Superdex 200 26/60 column. The fractions with PA200 from the Superdex column were loaded on a Uno Q column (6 ml, Bio-Rad), washed with 20 ml of the TSDG buffer containing 125 mM of KCl, and eluted with a 100-ml linear gradient of 125 to 500 mM KCl in the TSDG buffer. The PA200 pooled from the Uno Q step was finally separated by ultracentrifugation (85,000*g* for 19 h) on a 5% to 20% glycerol gradient.

### Histone purification and acetylation

Histones from rabbit thymus or HeLa cells were purified according to standard acid extraction protocols. As described (4), histones from thymus were acetylated by His-tagged Gcn5 HAT domain (aa 98–262) in the buffer containing 50 mM of Hepes, pH 8.0, 10% glycerol, 1 mM of DTT, 10 mM of sodium butyrate, and 0.3 mM of acetylCoA, and the reaction was terminated by TCA precipitation.

### Degradation of acetylated histone

The degradation of acetylated histones was assayed in the buffer containing 20 mM of Tris, pH 7.5, 0.5 mM of EDTA, 1 mM of DTT, and 1 mM of MgCl_2_ at 37 °C, and a 70-μl reaction mix was supplemented with 280 ng of the 20S proteasome and 3 μg of acetylated histones in the absence or presence of 1 μg of PA200.

### Quantification and statistical analysis

Unless stated otherwise, significance levels for comparisons between two groups were determined by two-tailed unpaired *t* test, mean and SEM (∗*p* < 0.05 and ∗∗*p* < 0.01), normal distribution. All of the images were chosen blindingly and randomly and quantitated by ImageJ.

## Data availability

The data that support the findings of this study are available from the authors upon request; correspondence and requests for materials should be addressed to X. -B. Q. (xqiu@bnu.edu.cn). We did not use any computer code in this article.

## Conflict of interest

The authors declare that they have no conflicts of interest with the contents of this article.
