# Supplementary material for: Proteasome subunit α4s is essential for formation of spermatoproteasomes and histone degradation during meiotic DNA repair in spermatocytes
Source: J Biol Chem. 2020 Dec 4;296:100130. doi: 10.1074/jbc.RA120.016485 (PMC7949063; doi:10.1074/jbc.RA120.016485)

**Proteasome subunit α4s is essential for formation of spermatoproteasomes and histone degradation during meiotic DNA repair in spermatocytes**

Zi-Hui Zhang^1^, Tian-Xia Jiang^1,*^, Lian-Bin Chen^1^, Wenhui Zhou^2^, Yixun Liu^3^, Fei Gao^3^,

and Xiao-Bo Qiu^1,*^

**Supplementary information**

**Supplementary figure legends**

**Supplementary Figure 1. Abnormal features are not detectable in spleen, kidney, and Sertoli cells in the α4s-deficient mice**

A, Schematic representation of the retroviral gene trap vectors for constructing α4s-deficient mice. LTR, long terminal repeat; Frt and F3, heterotypic target sequences for the FLPe recombinase; loxP and lox5171, heterotypic target sequences for the Cre-recombinase; SA, splice acceptor; βgeo, β-galactosidaseneomycin phosphotransferase fusion gene; pA, bovine growth hormone polyadenylation sequence. B, Fertility test of α4s^-/-^ males and females. Adult males of α4s^+/-^ or α4s^-/-^ genetic background were mated to wild type females at 2 months of age or older. The number of litters and pups was recorded. None of females that bred to α4s^-/-^ males was pregnant. α4s^-/-^ females exhibited no signs of infertility, producing similar sized litters as their heterozygous littermates. C, HE staining for spleen and kidney of the wild-type or the α4s-deficient mice at 84-day old. Scale bar: 1000 μm. D, Flow cytometric analysis of testicular cells. DNA content exclusion based on “Hoechst Blue” fluorescence. Spermatocytes with 4C DNA from WT and α4s-/- testis were collected, respectively. Haploid cells with 1C DNA content are collected as a control. E, Immunostaining for SOX9 in the wild-type or the α4s-deficient mice at 84-day old. Scale bar: 50 μm. Data are representative of one experiment with at least two independent biological replicates.

**Supplementary Figure 2. Deletion of α4s specifically reduces the proteasome activity in adult testes**

A, Peptidase activities and immunoblotting following native PAGE analysis of in the extracts from the testes of the wild-type or the α4s-deficient mice at 23 or 84 days old. Proteasomes were visualized by incubating with LLVY-amc in the absence or presence of 0.02% SDS. B, The proteasomal peptidase activities of testicle extracts of the wild-type or the α4s-deficient mice at different ages. Data are representative of one experiment with at least two independent biological replicates. **P* < 0.05, ***P* < 0.01 (two-tailed unpaired *t*-test).

**Supplementary Figure 3. Deletion of α4s increases the rate of apoptosis in spermatocytes**

A, Immunofluorescent staining for annexin V in the paraffin sections of the testes of the wild-type and the α4s-deficient mice at pnd 84. Scale bar: 40 μm. B, Percentage of annexin V -positive cells were analyzed (mean ± SEM, n=6). **P* < 0.05, ***P* < 0.01 (two-tailed unpaired *t*-test). Data are representative of one experiment with at least two independent biological replicates. C, Immunostaining of the spermatocyte nuclei from the wild-type or the α4s-deficient mice with SYCP1 and SYCP3. The white arrow points to sex chromosomes. Scale bar: 25 μm.

**Supplementary Figure 4. Deletion of α4s increases the number of the γ-H2AX-positive cells in testes**

A, Immunostaining of the testicle sections of the wild-type or the α4s-deficient mice with γ-H2AX. γ-H2AX-positive spermatocytes were quantitated. Scale bar: 150 μm. B, Immunostaining of the spermatocyte nuclei from the wild-type or the α4s-deficient mice. γ-H2AX foci on chromosomes were quantitated (n=20). Scale bar: 20 μm. C, Deletion of α4s interrupts the meiotic sex chromosome transcriptional silencing. All mice were 84-day old. Data are representative of one experiment with at least two independent biological replicates.

**Supplementary Figure 5.** **Deletion of α4s does not stimulate the recruitment of MLH1 in the nuclei of spermatocytes**

A, Immunostaining of the spermatocyte nuclei from the wild-type or the α4s-deficient mice at various ages. B, MLH1 foci on chromosomes in (A) were quantitated (n=20). Scale bar: 10 μm. Data are representative of one experiment with at least two independent biological replicates.

**Supplementary Figure 6. Deletion of α4s does not enhance the recruitment of RAD51 in the nuclei of spermatocytes**

A, Immunostaining of the spermatocyte nuclei from the wild-type or the α4s-deficient mice at various ages. B, RAD51 foci on chromosomes in (A) were quantitated (n=20). Scale bar: 10 μm. Data are representative of one experiment with at least two independent biological replicates. C-D, Immunostaining of the spermatocyte nuclei from the wild-type or the α4s-deficient mice at pnd 84. White arrows point to TUNEL foci on chromosomes (C), which were quantitated (n=20) (D). Scale bar: 20 μm. E, Negative and positive controls for TUNEL assay in Fig. S6C. The negative control was not treated with Terminal Deoxynucleotidyl Transferase, and the positive control was treated with DNase I for 5 min. F, Immunoblotting of the whole tissue extracts of the wild-type and the α4s-deficient testes from mice at pnd 84. Data are representative of one experiment with at least two independent biological replicates (mean ± SEM, n=6). *P < 0.05, **P < 0.01 (two-tailed unpaired t-test).

**Supplementary Figure 7. α4s is required for degradation of the acetylated core histones in testes**

A, Immunoblotting analysis of the extracts from the 4C spermatocytes of the wild-type and the α4s-deficient mice was performed as in Fig. 5D. The levels of H4K16ac were quantitated. B, The acetylated histones (Ac-H) were analyzed by immunoblotting following the acetylation-dependent degradation assay for the core histones by the testis lysates from the wild-type (WT) or the α4s-deficient (α4s−/−) mature mice. A 90 μl reaction mix was supplemented with 100 μg proteins in the testis lysates, and 6 μg of the acetylated core histones. Data are representative of one experiment with at least two independent biological replicates. *P < 0.05 (two-tailed unpaired t-test).


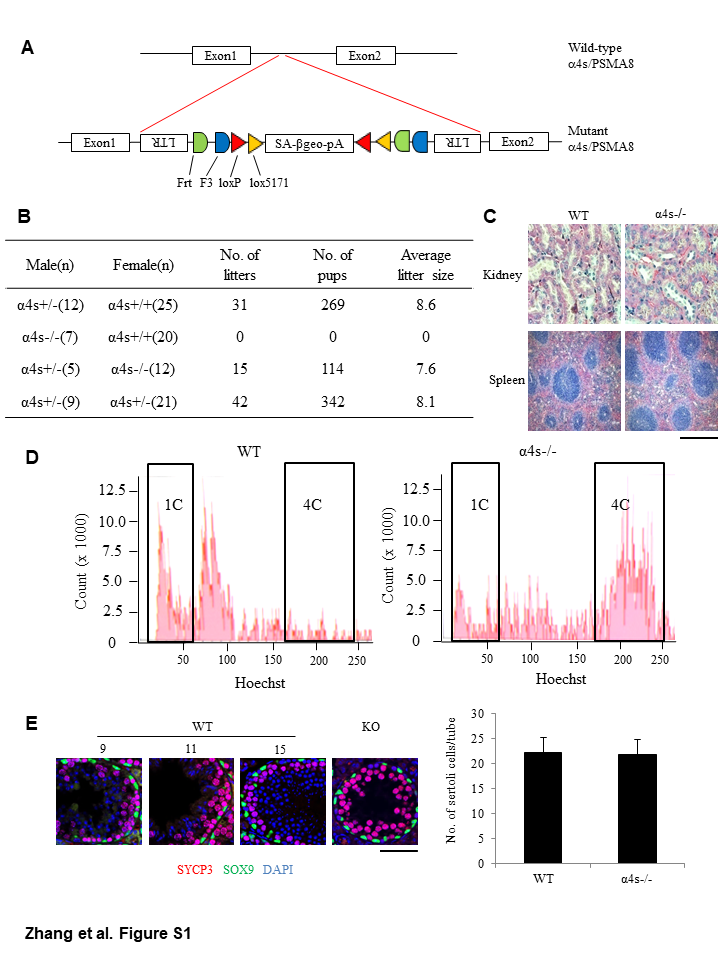


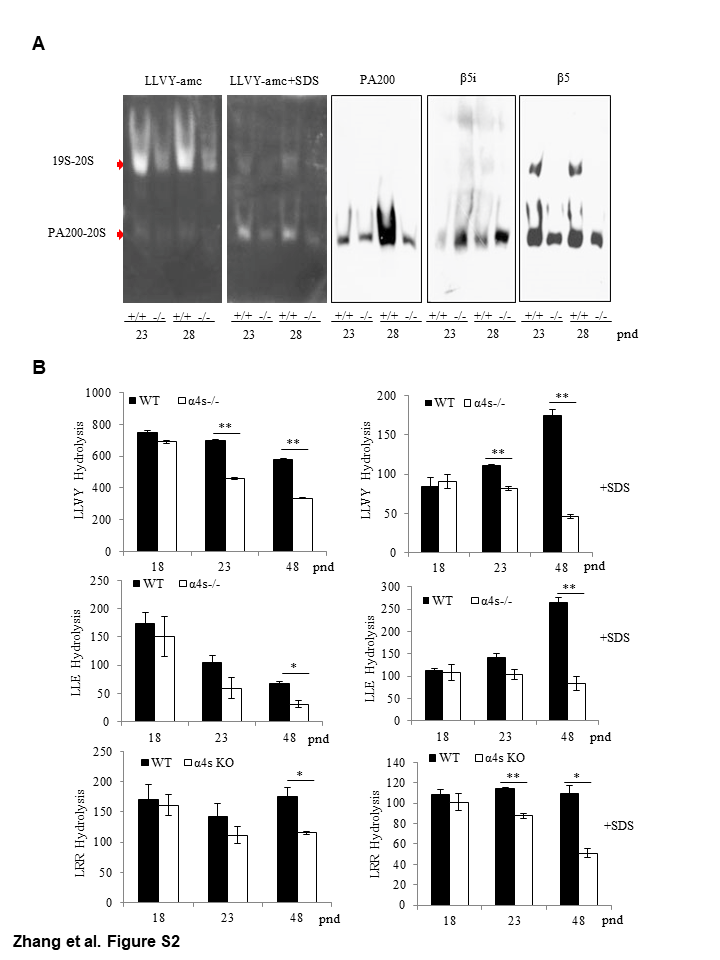


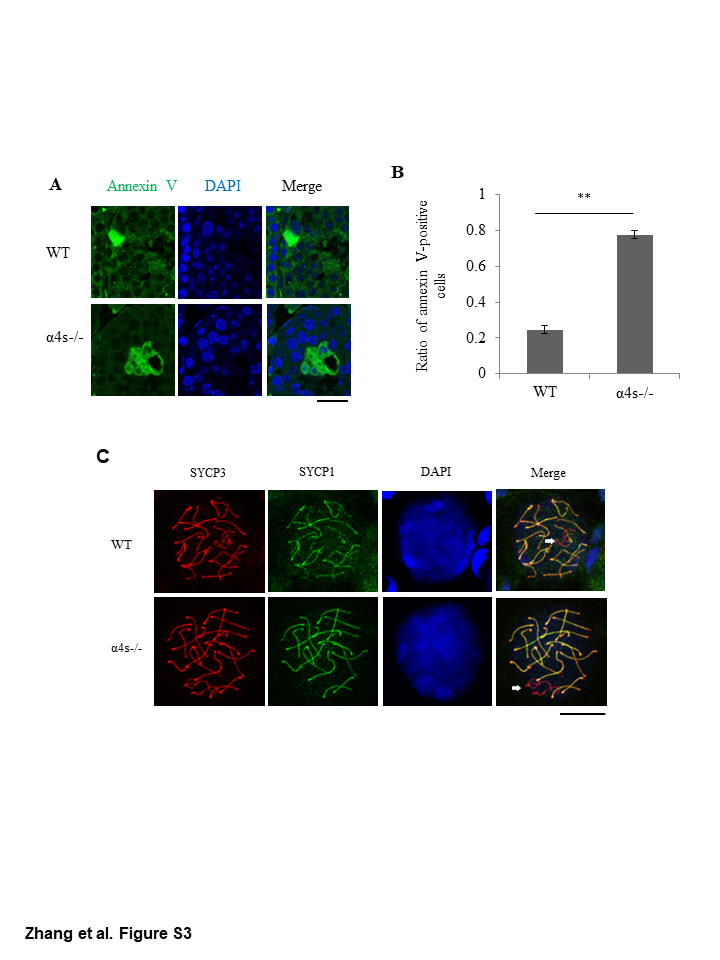


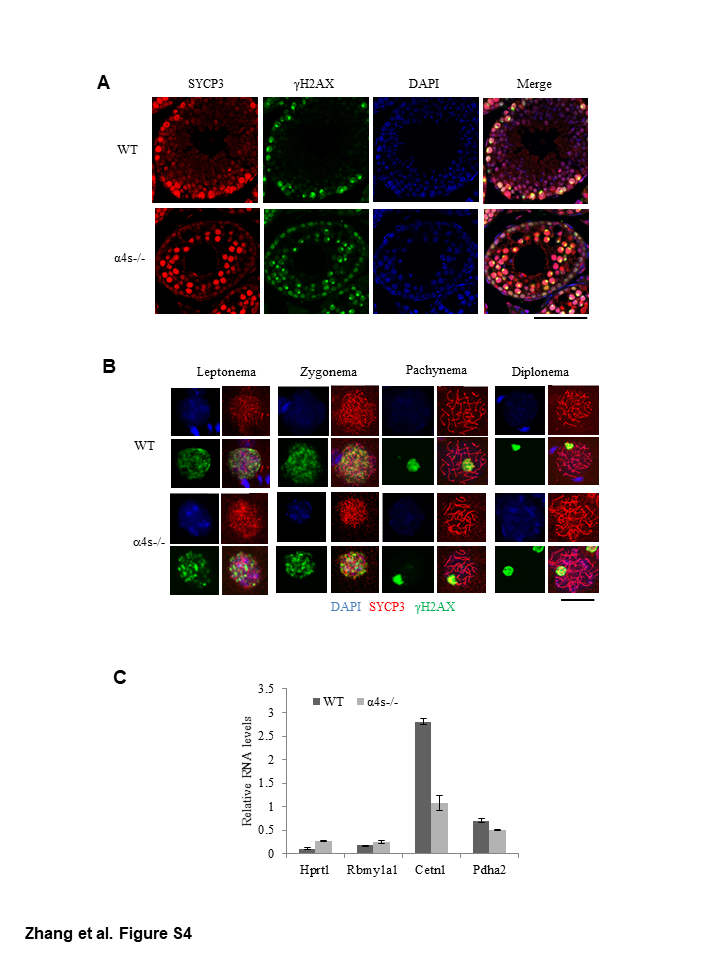


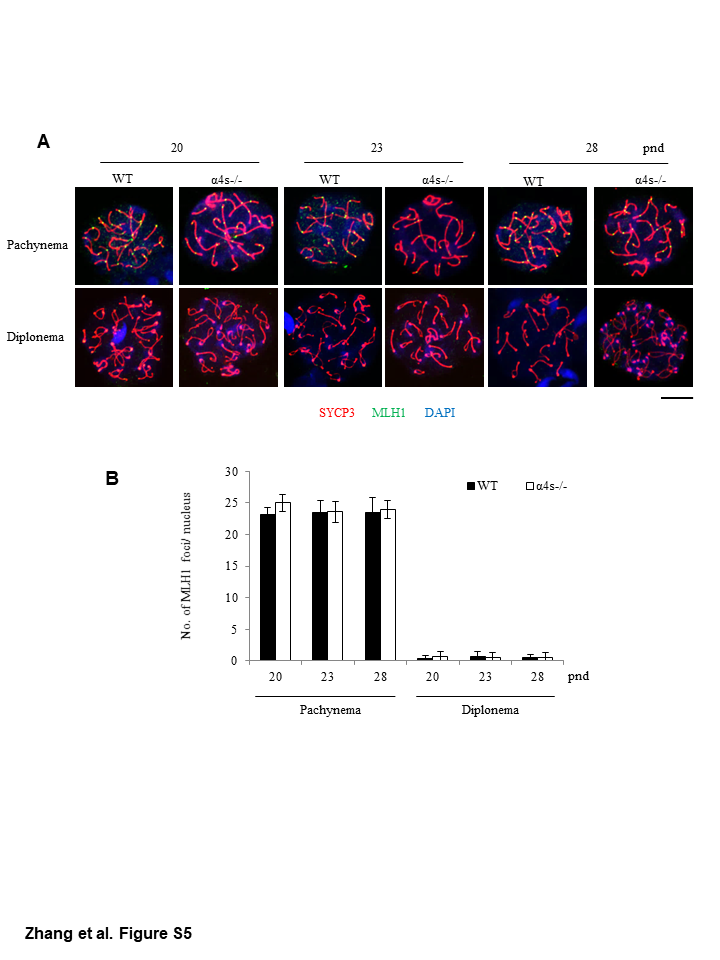


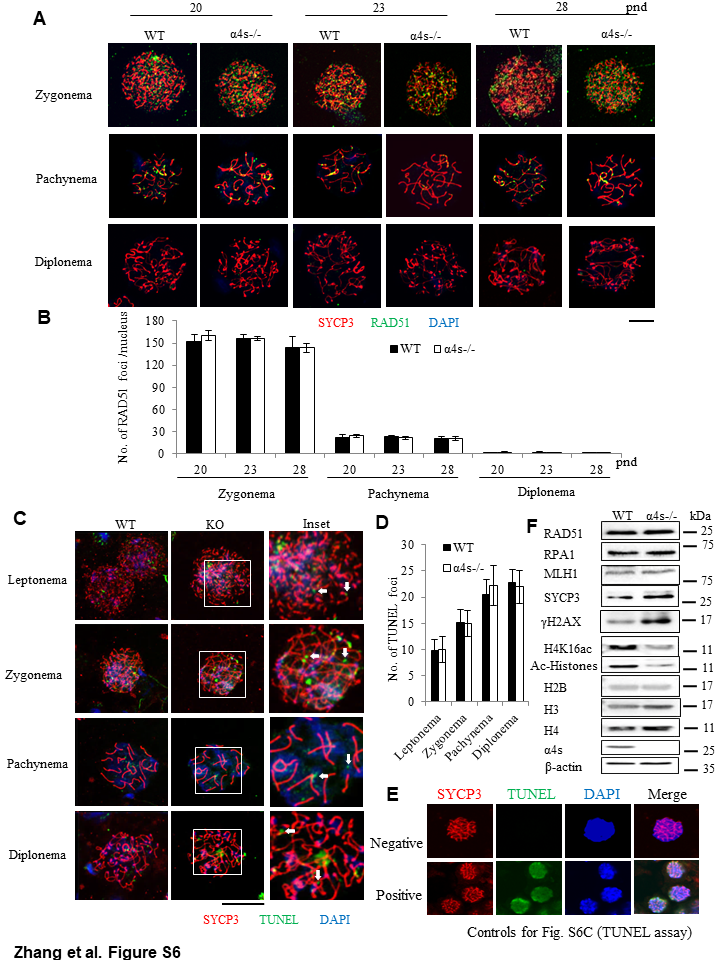


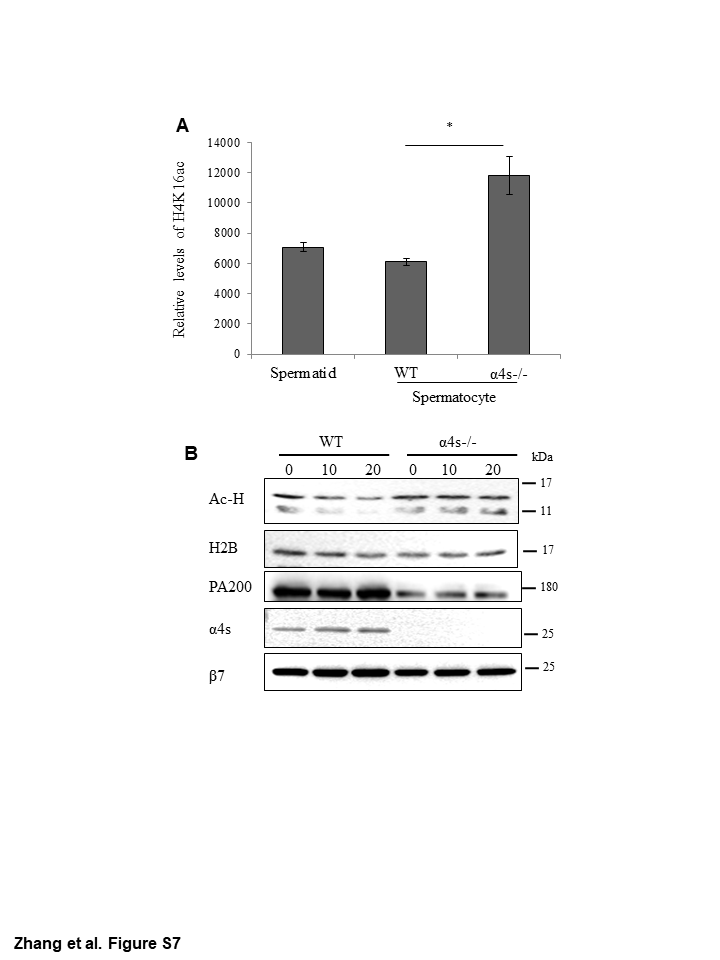

Supplement: Figures S1–S7 [file mmc1.docx]
